# Recent Advances in Genome-Engineering Strategies

**DOI:** 10.3390/genes14010129

**Published:** 2023-01-02

**Authors:** Michaela A. Boti, Konstantina Athanasopoulou, Panagiotis G. Adamopoulos, Diamantis C. Sideris, Andreas Scorilas

**Affiliations:** Department of Biochemistry and Molecular Biology, Faculty of Biology, National and Kapodistrian University of Athens, 15701 Athens, Greece

**Keywords:** CRISPR-Cas systems, dCas9, nucleases, ZFNs, TALENs, CAR-T cells, gene therapy, gene knockout, genome editing

## Abstract

In October 2020, the chemistry Nobel Prize was awarded to Emmanuelle Charpentier and Jennifer A. Doudna for the discovery of a new promising genome-editing tool: the genetic scissors of CRISPR-Cas9. The identification of CRISPR arrays and the subsequent identification of *cas* genes, which together represent an adaptive immunological system that exists not only in bacteria but also in archaea, led to the development of diverse strategies used for precise DNA editing, providing new insights in basic research and in clinical practice. Due to their advantageous features, the CRISPR-Cas systems are already employed in several biological and medical research fields as the most suitable technique for genome engineering. In this review, we aim to describe the CRISPR-Cas systems that have been identified among prokaryotic organisms and engineered for genome manipulation studies. Furthermore, a comprehensive comparison between the innovative CRISPR-Cas methodology and the previously utilized ZFN and TALEN editing nucleases is also discussed. Ultimately, we highlight the contribution of CRISPR-Cas methodology in modern biomedicine and the current plethora of available applications for gene KO, repression and/or overexpression, as well as their potential implementation in therapeutical strategies that aim to improve patients’ quality of life.

## 1. Introduction

In October 2020, the chemistry Nobel Prize was awarded to Emmanuelle Charpentier and Jennifer A. Doudna for the discovery of a new promising genome-editing tool: the genetic scissors of CRISPR-Cas9 [1]. The introduction of the present technology led to the flourishing of molecular biology and biotechnology, and a post-genomic era started. Using the CRISPR-Cas9 system, the DNA of eukaryotic organisms as well as microorganisms can be modified in a high-precision manner and efficiency, allowing for more thorough genetic and epigenetic studies [2,3,4].

The discovery of an array of special repeat sequences in 1987 by Ishino et al. during the study of the *Escherichia coli* (*E. coli*) genome [5], and the subsequent identification of *cas* genes a few years later, was the first step for the development of genome-engineering approaches for the manipulation of nucleic acids [2,6,7,8]. This array of special repeat sequences, named Clustered Regularly Interspaced Short Palindromic Repeats (CRISPR), along with CRISPR-associated (Cas) proteins, represent an adaptive immune surveillance process widespread across archaea as well as bacteria. This process causes interference against foreign nucleic acids derived from infectious organisms, such as phage viruses, and hence, being responsible for the protection of its holder [9,10,11,12,13,14,15].

A typical CRISPR locus consists of 21–47 bp DNA repeat sequences and non-repetitive nucleotides of similar size, usually 26–72 bp, called protospacers. Protospacers are DNA sequences originating from invading pathogens and, thus, represent a collection of genetic information stored in immunological memory [14]. In the genomic DNA sequence of interest, each protospacer is related to a specific sequence called protospacer adjacent motif (PAM), which can differ in each CRISPR system that is harnessed [16,17,18]. Genomic analysis of prokaryotic organisms with multiple CRISPR loci unveiled another structural characteristic located next to these short repeats, containing genes that encode diverse nucleases called Cas proteins, which are responsible for the scission of extrinsic DNA. The conserved region between the CRISPR locus and the *cas* genes is called leader and has an approximate length of 60 bp.

The process of CRISPR–Cas systems can be discriminated in three sequential steps: adaptation, expression and maturation, and interference. In the adaptation step, Cas proteins recognize the invader DNA and segmentate it, retaining a short DNA sequence that will function as a new protospacer after its integration into the CRISPR array. Two proteins, Cas1 and Cas2, which exist in most CRISPR-Cas systems, enable the incorporation of the spacers to the CRISPR cassettes [19]. The integration of each protospacer will define the immunological memory. In the step of expression and maturation, the transcription of the CRISPR array takes place. As a result, a precursor RNA transcript is generated and is subsequently divided into small RNA units, called CRISPR RNAs (crRNAs). This process is mediated by an RNA endonuclease complex or by an alternative mechanism that is mainly based on the bacterial RNase III [20]. Each crRNA is characterized by a single protospacer. Afterwards, the newly synthesized crRNAs interact with one or more Cas proteins and the active Cas–crRNAs complexes are generated [17,21]. Finally, in the interference step, the active Cas–crRNAs complex investigates the cell for extrinsic nucleic acids, based on the complementarity of the latter with the crRNAs. In the case of foreign DNA recognition, Cas–crRNAs complex cleaves the target DNA, protecting the cell from its attacker [22,23,24].

Although the manipulation of genomes has been employed by the utilization of previous genome-engineering approaches, like zinc finger nuclease (ZFN) [25] and transcription activator-like effector nuclease (TALEN) methodologies [26], the emergence of CRISPR-Cas technologies changed the way researchers study gene regulation and enabled researchers to modify gene expression in diverse levels, thus deciphering the hidden aspects of their functions. In this work, we describe the current CRISPR-Cas approaches used for genome engineering and discuss their applications in molecular biology, both in research and clinical practice. Furthermore, an extensive description of additional genome-engineering strategies, including ZFN and TALEN methods, is also discussed enabling a thorough comparison between previously introduced genome-editing technologies and the cutting-edge CRISPR-Cas methodology.

## 2. Diverse CRISPR-Cas Systems

Two classes (class I&II) of CRISPR-Cas systems have already been characterized across bacterial and archaeal cells, which are differentiated according to the function and structure of Cas effectors that take part in the procedure [27]. In particular, the systems of Class I are characterized by multiple complexes that are formed by the complicity of various Cas effectors, whereas class II systems exploit a single effector Cas protein. Class I and II systems can be further subdivided into six types (types I–VI). Class I systems include types I, III, and IV, whereas class II systems comprise the three remaining types (II, V, and VI) (Table 1).

### 2.1. Class I Systems

Type I CRISPR-Cas systems harness several Cas proteins that all interact together, generating a complex named CRISPR-associated complex for antiviral defense (Cascade). This term was initially utilized to describe the multi-Cas effector complex of *E. coli*, which was the first identified type I CRISPR–Cas complex [17]. However, the subsequent identification of several other type I system complexes that are extremely similar to *E. coli* Cascade, lead to the use of this term for every such complex. Additionally, type I systems feature *cas3*, a signature gene that encodes a protein, which demonstrates an ATP-independent nuclease activity for single-stranded DNA templates as well as an ATP-dependent helicase (Table 1) [28,29,30].

All type III CRISPR-Cas systems feature the signature *cas10* gene that encodes a protein with several domains able to target DNA [14]. Cas10 represents the large subunit of the effector complexes identified in these systems. In most cases, Type III CRISPR-Cas systems exploit crRNAs transcribed from CRISPR arrays associated with type I or type II systems and, hence, do not use proteins encoded by their *cas1* and *cas2* genes [21]. To date, there are four type III subtypes: III-A to III-D. Subtype III-A loci include *cas1*, *cas2*, and *cas6* [31], and are responsible for targeting foreign DNA [16]. On the contrary, these genes are absent in most III-B systems, whose action depends on other co-existing CRISPR-Cas effectors. In contrast to III-A systems that target only DNA, III-B systems can also target RNA [21,32,33]. The III-C subtype involves a Cas10 protein featuring an inactivated cyclase domain, whereas III-D systems feature a Cas10 protein that lacks histidine–aspartate (HD) domain.

Although the function and mechanism of the known CRISPR-Cas types have already been clarified, type IV systems remain unclear. These systems are primarily encoded by plasmids, suggesting a plasmid–host interaction for the repression of the host defense by resident CRISPR-Cas systems. Currently, type IV CRISPR-Cas loci are categorized into two subtypes, namely IV-A and IV-B. Each of these two subtypes share a specific group of effector module proteins [27]. Even though type IV *cas* operons are often related to CRISPR arrays, they lack specific key features of other CRISPR-Cas systems, such as the adaptation module and an effector. Thus, it has been suggested that these systems demonstrate differentiated CRISPR-Cas functions or are even functionally defective [34].

### 2.2. Class II Systems

Missing in archaea, type II CRISPR-Cas systems are present in ∼5% of the bacterial genomes, being over-represented among pathogens. The functionality of type II systems is based on the *cas9* gene, which leads to the expression of a multidomain protein that has the capability to target and cleave extrinsic DNA [14]. Two nuclease domains of Cas9 have been identified, namely HNH and RuvC. Both domains are required for the cleavage of the foreign DNA [35,36,37,38,39]. Apart from *cas9*, every type II CRISPR-Cas locus also contains *cas1* and *cas2* genes. Most of these systems encompass one or two genes, the transcription of which leads to the generation of a specialized RNA molecule, denoted as tracrRNA. Unlike most of the type I and III systems that harness a Cas endoribonuclease derived from the CRISPR-Cas loci to cleave the CRISPR precursor transcript (pre-crRNA), type II systems rely on the use of an endogenous RNase III and the tracrRNA [35,40,41,42,43]. Specifically, the procedure initiates with the base-pairing of tracrRNA with the pre-crRNA repeats in the presence of Cas9 to form RNA duplexes that are cleaved by the endogenous RNase III [20]. Subsequently, the existing crRNA is further processed, leading to the generation of mature crRNAs that remain duplexed with tracrRNA in Cas9-complex manner [2,20].

Like the previously described CRISPR-Cas systems, type V systems consist of three parts: the effector protein, the acquisition module, and the CRISPR array. When mobile genetic elements (MGEs) invade the cell of the host, Cas1 and Cas2 of the adaptation module create a complex to intercept a short sequence of the invasive molecule, the protospacer, next to the PAM of the CRISPR array. Subsequently, the array is transcribed to generate pre-crRNA, which is further processed by RNase III or effector proteins in its own CRISPR system to form mature crRNA. To date, most of the type V CRISPR-Cas-identified systems exhibit targeted RNA-guided cleavage activity of dsDNA substrates.

Three known subtypes of type V systems, subtypes V-A, V-B, and V-E, have been studied in detail, providing sufficient information regarding their mechanism of action. Cas12a, Cas12b, and Cas12e are the effector molecules of types V-A, V-B, and V-E, respectively. After the corresponding effector protein binds to the gRNA, the derived complex that is created recognizes the 5′ T-rich PAM motif and mediates the unwinding of the target DNA and its subsequent base pairing with the crRNA’s guide sequence. Concurrently, an “R-loop” is created by the displacement of the non-target strand of the target sequence. Then, RuvC domain cuts both strands successfully at PAM-distant sites, leading to an incision that is characterized by 5′ overhangs. Notably, there are major differences from the type II CRISPR enzyme Cas9, which acts on the PAM-proximal bond to generate blind ends. Except for targeting and cleaving dsDNA, some effector molecules of type V systems can use either dsRNA or ssRNA as substrate. Particularly, Cas12a, -b, -c, -d, -h, -i, and -j recognize and cleave ssDNA, whereas Cas12g targets ssRNA.

The recently identified RNA-targeting type VI CRISPR-Cas systems exclusively target ssRNA and are further divided into four main subtypes (A–D) [44,45,46]. The functionalities of subtypes VI-A, VI-B, and VI-D, along with their corresponding Cas13 effectors have already been elucidated. Cas13 effectors represent crRNA-guided RNases that are characterized by the presence of two distinct and independent catalytic centers. The first one processes the pre-crRNA, whereas the second catalytic center residing in the nucleotide-binding (HEPN) domain in higher eukaryotes and prokaryotes mediates the cleavage of ssRNA substrates. The cleavage preferentially takes place in exposed regions of the RNA secondary structures, mostly at uridine (U) or adenine (A) sites. Among the different microbial genomes that have been studied, it has been shown that the CRISPR-Cas loci of the VI-A, -B, and -D systems lack *cas1* and *cas2* genes; hence, they include only a single CRISPR array and a *cas13* gene encoding the effector protein. Even though adaptation modules are absent from these loci, most of these systems are derived from hosts possessing another CRISPR-Cas locus with *cas1* and *cas2* genes, indicating that these systems share some of their elements [47].

## 3. Utilizing Cas Nucleases as Genome-Engineering Tools

Due to the simplicity of class II CRISPR-Cas systems, in which a single Cas protein is sufficient to mediate the target’s binding and incision, they are easier to exploit for research purposes and have already been established as an efficient and powerful tool for genome editing approaches, both in prokaryotic and eukaryotic cells. Diverse technologies have been designed, each of which is suitable for a specific application, depending on the purpose of the scientific study [2,7]. Of note, protein engineering of CRISPR nucleases is mainly utilized to improve CRISPR as a method for genetic modification and manipulation, offering a variety of techniques for editing in multiple levels.

### 3.1. Cas9 Nuclease for Genome Editing

**Figure 1:**
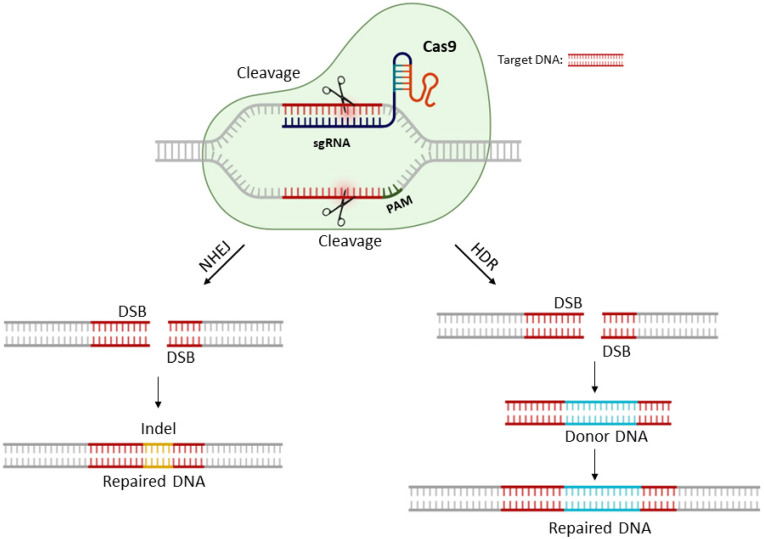
The CRISPR-Cas9 system mechanism on a target gene. The Cas9 protein is guided by a single-stranded RNA that is complementary to the site of interest, in a specific genomic region. The DNA is cleaved by Cas9, and the occurred DSBs can be repaired by two distinct repair pathways: the error-prone non-homologous ending joining (NHEJ), which will induce indels into the repaired DNA (left) and the homologous dependent repair (HDR) that requires a donor repair template that is incorporated in the target DNA sequence (right).

The most commonly used CRISPR nuclease for DNA editing is the RNA-guided Cas9 [48]. This protein promotes genome editing by stimulating double stranded breaks (DBS) at a target genomic locus, using a single-guide RNA (sgRNA) molecule. The sgRNA is a version of the naturally existing guide RNA complex (crRNA–tracrRNA complex) engineered into a single and continuous sequence. This molecule is harnessed to lead the Cas9 protein to bind and cleave both strands of a target sequence, causing DSB. Upon the cleavage by Cas9, the target locus is subjected to DNA damage repair, either via the error-prone non-homologous end joining (NHEJ) or the high-fidelity homologous direct repair (HDR) pathway. In the absence of any homologous template, the NHEJ pathway is activated, and the ends deriving from the DSB are rejoined, leaving scars in the form of insertion/deletion (indels) mutations. The NHEJ pathway can be harnessed to create gene KOs, as the indels occurred within the coding region of the target gene can lead to frameshift translations and premature stop codons [49]. The alternative DNA repair pathway, HDR, maintains the integrity of the repaired DNA sequence and is activated when a homologous piece of DNA is present in the nucleus. HDR can be leveraged for precise alterations at a target locus, provided that an exogenous repair template is utilized. The homologous repair template can be either a single-stranded DNA oligonucleotide (ssODN), or a double-stranded molecule with homology arms flanking the insertion sequence (Figure 1).

The former provides a simple and effective method for generating small modifications in the genome, including the single-nucleotide substitutions that can be exploited for the investigation of casual genetic variations [50]. Although the HDR mechanism is the most precise of the widely used methods for DNA repair, it is still characterized by decreased efficiency, especially for some human cell types, like induced pluripotent cells (iPSCs) or embryonic stem cells (ESCs). For that reason, several methodologies have been deployed to augment the efficiency of HDR, with the most common the inhibition of factors participating in NHEJ, and the enhancement of factors like CtIP and RAD18 in HDR. Respectively, repression of Ku70/80 or DNA ligase IV, which constitute key molecules of the NHEJ pathway, or even the tagging of Cas9 with minimal N-terminal fragment CtIP can stimulate HDR. Unlike NHEJ, studies have demonstrated that HDR pathway is mostly active in the dividing cells, and its efficiency depends not only on the type and state of the cells, but also on the genomic locus and the selected repair template [49].

**Figure 2:**
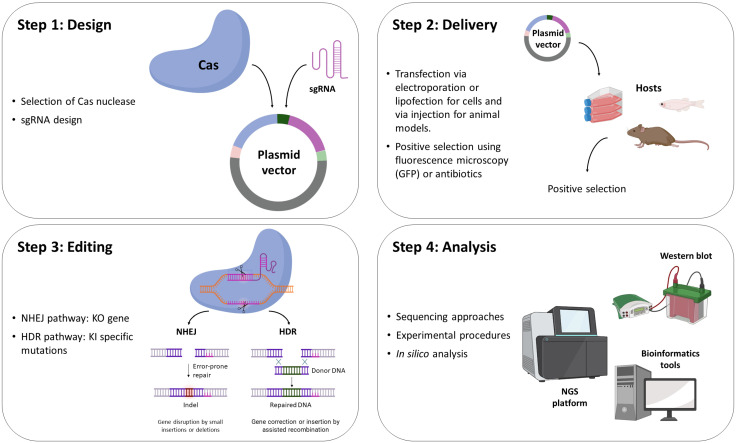
A typical workflow of a CRISPR-Cas9 editing strategy. The basic steps include the selection of the suitable Cas9 endonuclease and the design of the appropriate sgRNA molecule, their cloning into vectors and the subsequent delivery of the construct into eukaryotic cells to mediate changes into the target DNA. The final step corresponds to the experimental verification of the target DNA editing.

Since the characterization of the various CRISPR-Cas systems, a variety of Cas9 orthologs have been elucidated, and their usage as potential genome editing tools have been assessed. The most common sgRNA-guided endonuclease is SpCas9, originating from *Streptococcus pyogenes* [2,7,51]. This system represents a CRISPR-Cas approach that allows suppression of target genes, transcriptional repression or activation, single base-pair conversion, epigenetic modulations, and other manipulations of the genome (Figure 2).

SpCas9 recognizes a relatively simple PAM, 5′-NGG-3′, and mediates DSB a few bases upstream of the PAM sequence [2]. However, due to its large size (1368 amino acids), the *SpCas9* gene and its corresponding sgRNA molecule cannot be inserted together into specific viral vectors, such as adeno-associated virus (AAV) for their effective delivery into cells in vivo [52,53]. As an alternative, SaCas9 and CjCas9 orthologs can be used for genome editing when using AAV as cloning vectors. Due to their small size, SaCas9 (1053 amino acids) and especially CjCas9 (984 amino-acid residues) are more suitable options for conducting CRISPR-Cas9 methods using AAV vectors.

### 3.2. Cas12a Ortholog as a Lead Actor in DNA Editing

Although Cas12a and Cas9 have evolved through independent pathways, these endonucleases exhibit functional and structural similarities. To date, several studies have probed the utilization of Cas12a in DNA manipulation for several cell types, making the CRISPR-Cas12a system an alternative molecular genome editing tool [54]. Like in CRISPR-Cas9 systems, Cas12a produces a DSB at a specific genomic locus, and the subsequent activation of the host-cell repair machinery engages in the mending of the occurred DSB, either via NHEJ or HDR. The pre-crRNA processing activity of Cas12a makes this protein an attractive choice in cases of multiple gene regulation, which in the case of Cas9 is extremely challenging (Table 2) [55]. This auto-processing of its own crRNA has been exploited to alter many genetic elements at the same time, producing constitutive, inducible, and multiplexed genome engineering by the delivery of a single plasmid containing multiple CRISPR gRNAs. Aside from the impressive feature of Cas12a to perform high-specific dsDNA cleavage, it also demonstrates universal activity for ssDNA degradation upon activation with a ssDNA complementary to the crRNA guide, giving the opportunity for manipulating all DNA types [56].

### 3.3. Engineered Cas Proteins for Precise Editing

**Figure 3:**
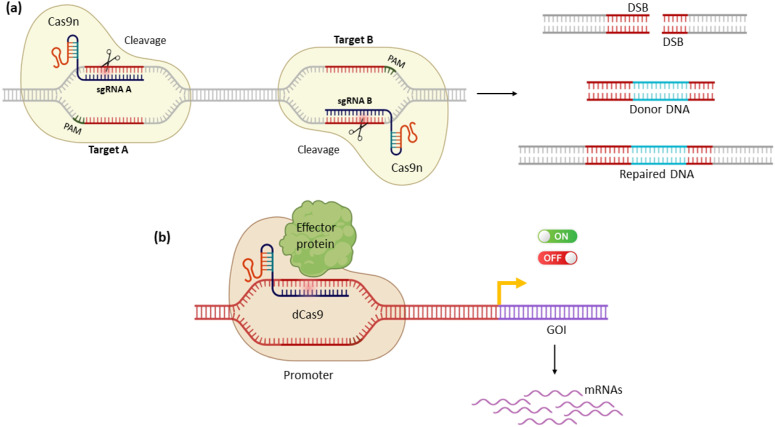
Schematic demonstration of the engineered CRISPR-Cas9 systems. (**a**) The nuclease domains of the Cas9 protein can be mutated independently to generate DNA nickases (Cas9n) that are able to introduce nicks, rather than DBS. DSBs can be introduced through the usage of a pair of sgRNA–Cas9n complexes. (**b**) The Cas9 protein is engineered into catalytically inactive Cas9 (dCas9). The dCas9 can be fused with specific effector proteins to mediate expression alterations of the gene of interest (GOI).

Several Cas9 and Cas12 nucleases with decreased off-target activity have been engineered while maintaining wild-type levels of on-target activity. Structure-guided mutagenesis of Cas9 catalytic domains led to the generation of engineered Cas9 proteins exploited for additional functions [2]. More precisely, an aspartate-to-alanine mutation in either RuvC catalytic domain (D10A) or a histidine-to-alanine change in HNH domain (H840A) led to the introduction of Cas9 “nicking” enzymes, named Cas9n, which perform single-stranded nicks rather than DSBs, reducing the potential off-target effects of the wild-type Cas9 [57,58]. The D10A mutation inactivates RuvC domain resulting in the cleavage of the target strand only, while H840A mutation causes the inactivation of HNH domain and leads to the generation of a non-target strand-cleaving nickase. Of note, Cas9n requires two sgRNA for double nicking, each one targeting its complementary DNA strand (Figure 3a). Since nicks are generally repaired with higher precision in eukaryotic cells, Cas9n can be used to create highly specific genome editing via HDR [7]. As opposed to the wild-type Cas9 that produces blunt DSB, Cas9n creates 5′ or 3′ overhangs along the target [8].

Introducing point mutations into both RuvC and HNH domains (D10A and H840A, accordingly) blocks the nucleolytic activity of Cas9, without affecting its binding affinity and capacity to the target DNA [59]. The generated mutant protein, called dead Cas9 (dCas9), has significantly further broadened the targeting range of Cas9 nucleases. Utilizing a sequence-specific nuclease as a molecule carrying out the delivery of other effector proteins to a specific locus in genome can introduce new DNA alteration capabilities. More precisely, fusing other functionally active protein domains to a dCas9 protein led to the generation of chimeric dCas proteins, capable of exhibiting the function of the effector protein to the specific DNA regions (Figure 3b) [60]. This methodology led to the newly introduced CRISPR technologies known as CRISPR activation (CRISPRa) and CRISPR interference (CRISPRi), respectively. Finally, dCas proteins can be fused with other effector proteins as well, including domains for investigating chromatin structure and three-dimensional chromatin, base-editing enzymes (cytidine or adenine deaminases) used to modify DNA, correct genetic mutations, or KO genes [61,62,63,64,65].

As for the latter, site-specific base editors have emerged as valuable tools for the correction of specific mutations associated with disease phenotypes or the introduction of mutations to suppress or alter the function of specific genes, giving new insights to therapeutic approaches corresponding to these diseases [66]. For example, dCas-guided base editors correct SNPs associated with hereditary diseases, such as thalassemia [67,68], Marfan syndrome [69], and phenylketonuria [70]. Furthermore, diverse approaches have been introduced for base-editing of DNA. In particular, the CRISPR-SKIP method is used for introducing point mutations into splice acceptor sites, resulting in exon skipping events. The subsequent translation of the generated modified mRNA will lead to the production of novel protein isoforms with altered features [71]. Another characteristic approach developed by fusing dCas9 with base editors is the CRISPR-Pass method, in which correction of nonsense mutations by adenosine editors takes place. CRISPR-Pass approach can be utilized as antiviral tool, capable of mutating viral genomes to block replication and protein synthesis of viruses including HIV, HBV, human papilloma virus, and Epstein–Barr virus [72].

Undoubtedly, the contribution of Cas orthologs into the genome engineering toolkit has enhanced efforts for genome editing, enabling the molecular cleavage of various genomic targets. However, the recognition of specific PAM sequences by Cas9 remains a barrier for the target selection, since these motifs limit the DNA regions that are suitable for editing. The engineering of Cas9 orthologs has led to the production of new Cas9 variants with altered features, which can be utilized to expand the targeting range of the genome. More specifically, Cas9-NG represents an engineered Cas9 variant that recognizes NGN PAMs, enabling the targeting of more genome loci. The ability of Cas9-NG to recognize PAMs in a less strict way allows us to edit genomes efficiently. In the same manner, the catalytic activity of SpG Cas9 variant depends on the PAM sequence and displays a highly promising genome editing tool [73]. Additional systems including VQR- and EQR-Cas9 as well as xCas9 can recognize sites containing the NG PAM. Of note, the VQR-Cas9 genome editor is specific for the NGA motif, whereas xCas9 variant comprise a broad range of recognition PAM sites, such as NG, GAA, and GAT [74]. Conversely, VRER SpCas9 variant can cleave targets with a TGCG PAM sequence [75].

The flourishing CRISPR-Cas technologies are about to upgrade not only genome- and epigenome-editing studies, but also the functional study of crucial genes at an RNA level. Since the introduction of CRISPR-Cas systems, many diverse technologies have been developed for precise editing of DNA and RNA molecules. Of note, CRISPR-Cas RNA-editing tools that have emerged enabled us to modify gene expression by editing the corresponding mRNA molecules, without the need of altering the gene sequence [76,77]. The development of these technologies paved the way for a new era in genomics/epigenomics and transcriptomics/epitranscriptomics, which was sealed with the establishment of CRISPR-Cas approaches in basic research as well as in clinical use.

## 4. Other Genome/Transcriptome Editing Strategies

Apart from the innovative technology of CRISPR-Cas systems, three additional genome-editing nucleases, homing endonucleases (HEs), zinc-finger nucleases (ZFNs), and transcription activator like effector nucleases (TALENs), are also utilized to perform breaks in DNA [78]. HEs, also known as meganucleases, are encoded by homing endonuclease genes (HEGs), which constitute highly specific DNA-cleaving enzymes that recognize asymmetric long DNA sequences (~20–30 bps) and naturally have been reported in microorganisms. The mechanism is based on the expression of HEGs in living cells, which are embedded in a mobile element. The encoded enzyme cleaves the target of interest and creates a DSB which can be repaired via HDR or NHEJ resulting in gene KO mutation of insertion of exogenous DNA [79,80].

ZFNs, the first custom DNA nucleases, are programmable synthetic proteins that bind to specific DNA locations and utilize DNA endonucleases to create DSBs, thus facilitating genome engineering [25,81]. These breaks are repaired either by HDR or NHEJ pathways. These engineered enzymes are fusions of two domains: the zinc finger repeats, that generate arrays of six or more fingers which can recognize approximately 9–18 bp, and a DNA-cleavage region from the restriction endonuclease Fok1, naturally found in bacteria, which dimerizes to target and cleave DNA sites [82]. To continue, TALENs are proteins that have been found in plant-pathogenic bacteria of genus *Xanthomonas*, and their role is to activate plant genes and support virulence [25,78,83].

In the same manner, TALENs are restriction fusion enzymes that have the ability to cut specific DNA sequences (~14–20 bps) and include two crucial and distinct domains: the DNA binding and a catalytic region, which is similar to the DNA-cleavage domain of ZFNs. DNA sites of interest can be targeting and cleaved for either KO or knock in (KI) genes using TALENs [84]. Both methods require the insertion of the ZFN/TALEN sequence into plasmids, which are transfected into cells. The ZFN/TALEN sequence is transcribed and translated into proteins, which enter the nucleus and bind to DNA in order to cleave the target sequence. Additionally, repair enzymes can generate gene KO, or a synthetic DNA can be incorporated to produce a gene KI [81,85].

On the contrary, the revolutionizing technology of RNA interference (RNAi) completely differs from the nuclease-dependent genome editing strategies. Although genome editing nucleases facilitate DSBs in genomic regions, RNAi performs post-transcriptional gene silencing by cleaving mRNA molecules [86,87]. The mechanism was first identified in 1998 by exposing the model organism *Caenorhabditis elegans* to a dsRNA that resulted in gene silencing [88]. RNAi constitutes a conserved biological process in eukaryotes to directly control genes and usually works for viral defense. The system involves two important RNA types: the small interfering RNA (siRNA) and the microRNAs (miRNAs). Naturally, siRNAs are derived from longer dsRNAs, whereas microRNAs are produced by ds precursor microRNAs. Both molecules are approximately 21 nts long and interact with Dicer, an endonuclease protein that recognizes ds RNA molecules and cleaves them into short segments [89]. Moreover, these small RNAs bind to Argonauts and other proteins creating an RNA-induced silencing complex, known as RISC [90,91]. siRNAs have perfect complementarity to specific mRNA sites, thus guide RISC to their target in order to activate Argonauts to break down the ds mRNA sites that they have created [91]. In the case of microRNAs, which can also direct RISC to mRNAs, only a part of the microRNA sequence is complementary and pairs with the target. The imprecise matching of microRNAs enables targeting hundreds of molecules leading to mRNA degradation or translation inhibition [92,93].

## 5. CRISPR-Cas System Versus Other DNA/RNA Editing Methods

Genome editing flourished with the development of powerful bioengineering techniques, namely ZFNs, TALENs, and CRISPR-Cas, which allow permanent modifications at a specific genomic site on a target organism [81]. Previous DNA editing techniques were obtained via homologous recombination by delivering a DNA template with 5′- and 3′-homology arms to the targeted genomic region, and host nucleases were used to repair the DNA breaks. However, the approach proved to be time-consuming, and the designed construct requires the delivery of a long DNA template and is inefficient in various mammalian cells. Thus, the design of special nucleases, that can specifically recognize and cleave DNA targets, constitutes a real breakthrough in genome engineering that revolutionized biomedical research and clinical medicine [94].

Although ZFNs and TALENs are transformative tools that have expanded the ability to manipulate genes and organisms, both have limitations and harbor disadvantages that the CRISPR-Cas system aims to overcome [95,96]. In particular, the zinc finger motifs of ZFNs are aligned in an array which affects the specificity of the neighboring zinc fingers. Consequently, both the design and the selection of desired zinc finger domains are challenging and time-consuming, while the target specificity of the system is hardly predicted (Table 3). Similarly, TALENs are based on protein–DNA interactions, which influences the specificity of the method. On the contrary, the engineering guidelines of TALENs is more flexible and simpler than ZFNs, since each TALEN domain recognizes only one nucleotide having well-defined target specificities [97,98].

Among the gene editing techniques, the newest CRISPR-Cas technology provides several advantages, becoming one of the most promising tools for genome manipulation. More precisely, the in vivo, or in vitro, delivery of both ZFNs and TALENs can lead to toxicity or lethality due to binding at off-target sites that introduces breaks in undesired regions [79,97].

The CRISPR-Cas system is driven by RNA–DNA base pairing, avoiding protein–DNA interactions, and offering many advances over ZFNs and TALENs, including simpler design for any DNA target, easy handling, higher efficiency, limited off-target sites, lower cost, and the ability of altering different genomic sites at the same time by adding multiple gRNAs (Table 3). As far as transcriptome editing is concerned, comparing RNAi, the traditional post-transcriptional gene silencing mechanism, to the CRISPR-Cas system, the latter utilizes sgRNAs that can eliminate all the transcript variants of a gene with reduced off-target effects [79,98]. Undoubtedly, the superior capabilities of CRISPR-Cas systems promise to expand our knowledge about genome engineering and pave the way for extraordinary advances in the biomedical field.

## 6. Applications of CRISPR-Cas Systems

In just a few short years, CRISPR-Cas systems have gained ground in modern scientific research and life sciences and contributed to outstanding breakthroughs in biotechnology and modern medicine. This versatile genome editing tool has already been applied widely in several areas including agriculture, bioenergy, biotechnology, and medicine [99]. As far as agriculture is concerned, gene editing via CRISPR-Cas technology is of great importance since it can be employed to produce CRISPR-modified foods or the creation of disease-resistant and drought-resistant crops to reduce food waste and prolong the shelf-life of foods [100]. Additionally, the utilization of the CRISPR-Cas system can be valuable for bioenergy. For instance, gene editing techniques can be used to increase tolerance of yeast to harsh conditions during the production of biofuels. Moreover, utilizing CRISPR-Cas systems for KO genes of transcription factors that control creation of lipids in algae can lead to increased lipid production for biodiesel [101,102].

Notably, CRISPR-Cas technology has mostly been applied in modern biomedical research enabling a plethora of applications for manipulating genes, cell lines, and animal models, thus the technology has been adopted in multiple gene therapy approaches [103,104,105]. Editing animal models is significant for understanding human diseases and developing novel therapeutics. Especially, the innovative CRISPR-Cas approach can be used to generate a wide variety of transgenic animal models, including zebrafish, *C. elegans*, and murine, at different developmental stages (Table 4) [106].

Creating transgenic animals with CRISPR KO and KI for testing specific genes or gene panels can provide novel insights into cellular mechanisms that provoke human diseases, monitor disease progression, and predict the efficiency of drug therapies [107]. Additionally, CRISPR editing in animal models is the first step for the development of efficient gene therapies that are based on replacing mutant genes with wild type genes. For instance, CRISPR KI has been used to introduce the retinitis pigmentosa (RP) gene in mouse models to examine the potential of reverse blindness in mice [108,109]. Another approach to treat sickle-cell disease was reported in mouse models in which β-globin has been edited to increase fetal hemoglobin levels [110].

CRISPR-Cas systems have facilitated stem cell regulatory biology since they can be investigated at the genome-wide level. The first attempt was carried out in 2013 when CRISPR-Cas9 was applied to stem cell models from cystic fibrosis patients for the correction of *CFTR* gene mutation [111]. Thenceforth, the method has been applied in various stem cell models for the correction of genetic mutations related to human diseases such as beta thalassemia, hemophilia, and Fragile X Syndrome [112,113]. Additionally, the CRISPR-Cas9 system has been used to manipulate organoid cultures to study genetic defects in diseases. Indicatively, CRISPR-Cas9 is utilized to KO the polycystin1 and polycystin2 (*PKD1* and *PKD2*) genes of human embryonic stem cells and generated mutant kidney organoids with obviously abnormal features [114,115]. Moreover, CRISPR-Cas9 editing has also been employed to hematopoietic stem cells to study responses in HIV patients [116]. Specifically, a certain mutation in the chemokine co-receptor type-5 (*CCR5*) gene is known to be responsible for asymptomatic carrying of HIV. CRISPR-Cas9 has been utilized for the generation of HIV-resistant cells through the disruption of the *CCR5* gene in stem cells from HIV patients [117,118]. Additional studies have shown that the disruption of *CCR5* has no toxicity on cells, thus the edited HIV-resistant cells could effectively reconstitute the immune system and be protected from HIV infection [119]. In the same manner, bone marrow cells have been removed from patients with sickle cell disease, and CRISPR-Cas9 disabled the B-cell Lymphoma 11A (*BCL11A*) gene, which is responsible for changing γ-globin into the β-globin chain, resulting in an increase of the fetal hemoglobin production in red blood cells (Table 5) [120,121,122].

Utilizing CRISPR-Cas for creating cells with special features allows for an in-depth understanding of the developmental stages of cells or tissues that has undoubtedly enhanced the development of cell-based therapies. Particularly, using fusions of dCas9 and transcriptional mediating proteins that are guided by a specific RNA can be introduced to cells for targeting genes of interest and act as transcriptional activators or repressors [123]. For example, the engineered CRISPR-dCas9 system has been used for targeting the Granulin (*GRN*) gene, a growth factor that promotes tumor progression in liver cancer [124,125]. The introduction of the dCas9 fused with epigenetic suppressor genes, such as Krüppel-Associated Box Transcriptional Repression Domain (*KRAB*), DNA Methyltransferase (*DNMT3a*), and Histone 3 Lysine 27 Methyltransferase (*EZH2*), and gRNAs that target *GRN* leads to decreased levels of its mRNAs in Hep3B hepatoma cells (Table 4) [126]. Further studies that focus on bladder cancer have revealed that the dCas9 tethering with the *CRY2-CIB1* (Cryptochrome 2-Calcium and Integrin Binding 1) photosensitive module can activate the expression of p53 and E-cadherin proteins, inhibiting the biological function of T24 tumor cells [126,127]. Furthermore, T lymphocytes are human cells that have been genetically edited to express an artificial chimeric antigen receptor (CAR) that can recognize specific malignancies and activates the immune system to target and destroy cancerous cells [128]. The development of CRISPR-mediated genome editing approaches is considered a breakthrough for immunology and lays the groundwork for the improvement of CAR-T therapeutic strategies. Of note, the advances of CRISPR gene editing as compared to previously existing genome editing techniques will both increase the safety of CAR-T cell therapies and enhance the tumor-killing activity of CAR-T cells [129,130]. For instance, CRISPR-Cas system has been utilized for disrupting the PD-1 receptor which normally binds to its ligand, PD-L1, and inhibits the function of T-cells. It has been shown that CRISPR KO can limit the expression of PD-1 on the surface of the CAR-T cells which ultimately led to increased tumor-killing ability and cancer prevention (Table 5) [131,132].

## 7. Conclusions

The introduction of CRISPR-Cas technologies paved the way for the development of novel approaches that aim to facilitate genomic and epigenomic studies, providing us the ability to decipher in depth the function of genes as well as their regulator mechanisms. More specifically, the development of diverse methods grounded on CRISPR-Cas systems laid the groundwork for the study of the genetic information in multiple levels since these technologies enabled us to mediate KO in vital genes, alter the epigenomic profile of the DNA, and correct the sequence of mutated genes that are responsible for hereditary diseases. Of note, the attempt to utilize CRISPR-Cas systems in clinical practice proved promising since it gave new insights into therapeutics, suggesting that these systems could be exploited for the development of novel therapeutic approaches for many human diseases.

## Figures and Tables

**Table 1:** Identified CRISPR-Cas systems in procaryotes, their required elements, as well as the types of molecules each one targets.

Class	Type	Pre-crRNA Processing	Effector Protein(s)	Target Cleavage	Target
I	I	Cas6	Cascade complex (Cas8, Cas7, Cas5, Cas6, Cas11 and Cas3)	Cas3	dsDNA
	III	Cas6	Cas7, Cas5, Cas10	Cas10	dsDNA/ssRNA
	IV	Cas6	Cas7, Cas5, Csf1	-	dsDNA
II	II	RNA III/Cas9	Cas9	Cas9	dsDNA
	V	Cas12	Cas12	Cas12	dsDNA/ssDNA/ssRNA
	VI	Cas13	Cas13	Cas13	ssRNA

**Table 2:** Diverse Cas orthologs identified among the bacterial species that have been studied. Each one derives from a specific organism and is characterized by a certain PAM sequence.

Cas Ortholog	PAM Sequence	Organism Origin	Description
SpCas9	5′-NGG-3′	*Streptococcus pyogenes*	The widely known Cas9 ortholog used for CRISPR/Cas9 approaches.
CjCas9	5′-NNNNRYAC-3′	*Campylobacter jejuni*	The smallest Cas9 ortholog that has been identified. Ideal for adeno-associated virus delivery.
SaCas9	5′-NNGRRT-3′	*Staphylococcus aureus*	Ideal for adeno-associated virus delivery to somatic tissues due to its considerably smaller size.
NmeCas9	5′-NNNNGATT-3′	*Neisseria meningitidi*	Ideal for adeno-associated virus delivery to somatic tissues due to its considerably smaller size.
FnCas9	5′-HGG-3′	*Francisella novicida*	In contrast to SpCas9, RuvC-like nuclease domain of FnCas9 cleaves the non-complementary DNA strand 6–7 bp from the PAM.
ScCas9	5′-NNGT-3′	*Streptococcus canis*	Increases the number of DNA sequences that can be targeted with Cas9 gene editing
AsCas12a	5′-TTTV-3′	*Acidaminococcus sp.*	Features a RuvC endonuclease domain and a putative novel nuclease domain
LbCas12a	5′-TTTV-3′	*Lachnospiraceae bacterium*	Suitable for plant gene targeting (GT)
FnCas12a	5′-TTTV-3′, 5′-KYTV-3′, 5′-VTTV-3′	*Francisella novicida*	In contrast to SpCas9, FnCas12a cleaves target DNA in a staggered pattern and leaves 5′ overhangs after initiation of a DSB in DNA. Features the RuvC domain, but lacks a second endonuclease domain

R = A/G, V = G/A/C, Y = C/T.

**Table 3:** Main differences between HEs, ZFNs, TALENs, and CRISPR-Cas genome editing systems.

Features	HEs	ZFNs	TALENs	CRISPR-Cas
Origin	Mobile genetic elements in microbiome	Eukaryotic transcription factors	TALENs of plant pathogenic bacteria	Bacterial adaptive immune system
Target sequence (bp)	20–30	9–18	14–20	~23
Number of target sites	Limited	Various	Various	Various
Engineering	Simple	Difficult	Slightly difficult	Very simple
Size (kb)	~1	~1	~3	>3
Target recognition	Protein-DNA	Protein-DNA	Protein-DNA	RNA-DNA and Protein-DNA
Specificity/ Off-target effects	High/Low	Low/High	High/Low	Highest/Low to high
Targeting efficiency	Low to variable	Variable	Variable to high	High
Multiple targeting	No/Protein engineering for each new target	No/Protein engineering for each new target	No/Protein engineering for each new target	Yes/Multiple targets can be edited simultaneously

**Table 4:** Applications of the CRISPR/Cas9 system in animal models and human cell lines to study different cancer types.

Malignancy	Model/Cell Line	Genes of Interest
Brain cancer	Mice models	*TP53, PTEN, NF1*
Breast cancer		*PYCR1*
Ovarian cancer	Murine models	*TP53, BRCA2*
Lung cancer		*p107*
Pancreatic cancer	Mice models	*BRCA1, BRCA2, PTEN, ATM*
Leukemia		*BCR-ABL1*
Hepatocellular carcinoma	Zebrafish models	*AR*
Colorectal cancer	HInEpC	*APC, TP53, SMAD4*
Liver cancer	HepG2, Huh-7	*NCOA5, ASPH, BAX, BCL2, CXCR4, CDK7*

**Table 5:** Applications of the CRISPR/Cas9 system used in clinical trials.

Disease	Target Gene	Type of Edit	Phase
Beta-thalassemia	Hemoglobin Subunit Beta (*HBB*)	Gene correction	I & II
Beta-thalassemia	BAF Chromatin Remodeling Complex Subunit 11A (*BCL11A*)	Gene disruption	II & III
Transfusion-Dependent β-Thalassemia	BAF Chromatin Remodeling Complex Subunit 11A (*BCL11A*)	Gene disruption	III
Acute Lymphoblastic Leukemia	CD19/CD52 molecule (*CD19/CD52*), T-Cell Receptor alpha & beta locus (*TCRαβ*)	Gene KO	I
Acute Lymphoblastic Leukemia	T-Cell Receptor alpha locus (*TCRα*)	Gene KO	I & II
Acute Lymphoblastic Leukemia	T-Cell Receptor (*TRC*), anti-CD19 CAR	Gene KO & KI	I & II
Acute Myeloid Leukemia	CD38/CD33 molecule (*CD38/CD33*)	Gene KO	I & II
Hepatocellular Carcinoma	Programmed Cell Death 1 (*PD-1*)	Gene KO	I
B-Cell Malignancies	T-Cell Receptor (*TCR*) & Beta-2-Microglobulin (*B2M*)	Gene disruption	I & II
B-Cell Non-Hodgkin Lymphoma	CD19 molecule (*CD19*), Programmed Cell Death 1 (*PD-1*), T-Cell Receptor (*TCR*)	Gene KO/KI	I
Billiary Tract Cancer	TGF-β receptor (*TGFβR*)	Gene KO	I
Prostate Cancer	Programmed Cell Death 1 (*PD-1*)	Gene KO	I & II
Esophageal Cancer	Programmed Cell Death 1 (*PD-1*)	Gene KO	I
Gastro-Intestinal Cancer	Cytokine inducible SH2 containing protein (*CISH*)	Gene KO	I & II
Human papillomavirus related cervical cancer	Human papillomavirus types 16 & 18 (*HPV16* & *HPV18 E6/E7*)	Gene KO	I
Metastatic Non-small Cell Lung Cancer	Programmed Cell Death 1 (*PD-1*)	Gene disruption	I
Multiple Myeloma	T-Cell Receptor alpha & beta locus (*TCRαβ*) & Programmed Cell Death 1 (*PD-1*)	Gene KO	I
Nasopharyngeal Carcinoma	Programmed Cell Death 1 (*PD-1*)	Gene KO	I & II
Non-small Cell Lung Cancer	Programmed Cell Death 1 (*PD-1*)	Gene KO	I & II
Relapsed or Refractory B-cell malignancies	T-Cell Receptor alpha locus (*TCRα*), CD19/CD22/ CD52 molecule (*CD19/CD22/CD52*)	Gene KO	I
Renal Cell Carcinoma	Major Histocompatibility Complex Class I (*MHC-I*) & T-Cell Receptor (*TCR*)	Gene insertion/KO	I
Solid Tumors	KO of *TCRαβ*, & insertion of genes encoding chains of a neoantigen-specific TCR (*neoTCR*)	Gene insertion/KO	I
Solid tumors	CD38 molecule (*CD38*)	Gene KO	I
Herpes Simplex Virus Refractory Keratitis	Helicase-primase subunit/ Single-stranded DNA-binding protein (*UL8/UL29*)	Gene disruption	I & II
Leber Congenital Amaurosis	Centrosomal Protein 290 (*CEP290*)	Gene correction	I & II
Hereditary Angioedema	Kallikrein B1 (*KLKB1*)	Gene KO	I & II

## Data Availability

No new data was created or analyzed in this study. Data sharing is not applicable to this article.
